# Emerging 2D Materials with Nonparabolic Bands for Ultrafast Photonics

**DOI:** 10.1002/smsc.202300030

**Published:** 2023-09-11

**Authors:** Guanyu Liu, Zhengwei Cui, Fangteng Zhang, Youjun Zeng, Weicheng Chen, Yuxian Liu, Zhaogang Nie, Qiaoliang Bao

**Affiliations:** ^1^ School of Physics & Photoelectric Engineering Guangdong University of Technology Guangzhou 510650 China; ^2^ Guangdong-Hong Kong-Macao Joint Laboratory for Intelligent Micro-Nano Optoelectronic Technology School of Physics and Optoelectronic Engineering Foshan University Foshan 528000 China; ^3^ National Key Laboratory of Science and Technology on Micro/Nano Fabrication Institute of Microelectronics School of Electronics Engineering and Computer Science Peking University Beijing 100871 P. R. China; ^4^ Institute of Energy Materials Science (IEMS) University of Shanghai for Science and Technology Shanghai 200093 China; ^5^ Nanjing kLIGHT Laser Technology Co. Ltd. Nanjing Jiangsu 210032 China

**Keywords:** 2D materials, mode-locked, non-parabolic bands, ultrafast photonics

## Abstract

In recent years, 2D materials have been widely used in optical and photonic applications such as mode lockers, optical switches, polarizers, and optical modulators. As is known, many 2D optical materials are parabolic semiconductors. The electronic states determines the material's optical characteristics. The energy band structure with a bandgap dominates the electron dynamics process associated with intrinsic light absorption. Particularly, the Dirac cone and flat band (FB) are special nonparabolic energy band structures in materials. Dirac materials are characterized by a zero bandgap, and FB materials have a completely flat dispersionless band in the Brillouin zone. These materials are used as emergent and promising 2D materials for saturable absorbers (SAs) due to their excellent nonlinear optical response, and they become potential candidates for ultrafast photonics applications. Herein, the focus is on the characteristics of the energy band structure; the carrier dynamics are reviewed, and application in ultrafast photonics are summarized.

## Graphical abstract

Materials with nonparabolic bands focus on different applications in ultrafast photonics, such as ultrafast carrier dynamics and nonlinear optical response. Linear dispersive Dirac bands have massless electrons like a photon, while nondispersive flat bands host electrons with an infinite mass. Nonlinear optics research on these materials provides new insights into the optical properties of those unconventional materials.

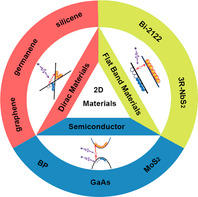

## 1 Introduction

Since the successful separation of atomic layer graphene by mechanical exfoliation in 2004,^[^ ^1^ ^]^ graphene‐like 2D materials have emerged as an important family of materials for fundamental research and applications. Beyond graphene, a variety of 2D materials have been successfully synthesized or separated from intrinsic materials, such as single‐element nanosheets (e.g., silicene,^[^ 2, 3 ^]^ germanene,^[^ 4, 5 ^]^ stanene,^[^ ^6^ ^]^ borophene^[^ ^7^ ^]^ and phosphorene,^[^ ^8^ ^]^ etc.), transition metal dichalcogenides (TMDs),^[^ ^9^ ^]^ main‐group metal dichalcogenides,^[^ 10, 11 ^]^ MXenes,^[^ ^12^ ^]^ etc. These materials cover superconductors, metals, semimetals, semiconductors, and insulators.^[^ 13, 14 ^]^ The energy band structure and electronic properties of these 2D materials are very different. Compared with the bulk materials, the surface atoms of a single‐layer 2D material are almost entirely exposed, resulting in a large surface area and strong quantum confinement. In this regard, many 2D materials show unprecedented optics, mechanics, heat, and magnetism performance. In the past ten years, photonics and nonlinear optics of 2D materials have attracted widespread attention^[^ 15, 16 ^]^ because most 2D materials have stronger light–matter interactions than intrinsic materials due to quantum confinement.^[^ ^17^ ^]^ Compared with other semiconductor materials, 2D materials have a unique 2D planar atomic structure, which makes their structure stable and has a larger surface area. 2D materials have excellent electronic and optical properties, such as good electron mobility, thickness‐dependent band structure, strong light absorption strength, and broadband absorption.^[^ ^18^ ^]^ The tunability and richness of band structure indicate that 2D materials have unique advantages and application prospects for optoelectronics.

**Figure 1:**
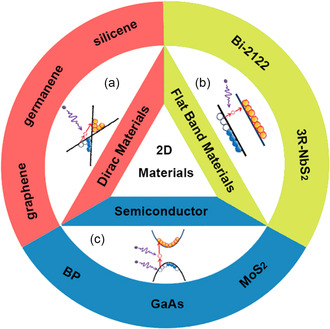
Emerging 2D material classifications: a) Dirac materials; b) FB materials; c) semiconductors.

2D materials have been widely studied for nonlinear optics due to their mechanical flexibility and compatibility with optical components.^[^ 15, 16 ^]^ In particular, the nonlinear optical (NLO) phenomenon, including saturable absorption, optical limiting, two‐photon absorption (TPA), and harmonic generation in the 2D materials, has been extensively studied.^[^ 19, 20, 21 ^]^ Traditional saturable absorbers (SAs) include organic chemical dyes, doped crystals, and semiconductor saturable absorption mirrors (SESAMs). Organic chemical dyes are toxic with poor stability. The doped crystal has a narrow absorption band with inherent defects. Although SESAM has a good modulation effect, the production cost is high due to the complex preparation process that depends on molecular beam epitaxy technology or metal–organic gas phase epitaxy technology. In addition, the wavelength tunability of SESAM is limited. Researchers have recently developed a series of 2D SAs such as graphene, topological insulators, and black phosphorus (BP) for mode‐locked or Q‐switch lasers. Due to the material diversity and structural characteristics, 2D SA generally has thickness‐dependent modulation depth, broad operation wavelength range, fast relaxation time, and good mechanical flexibility. Furthermore, the preparation of 2D SAs relies on a chemical vapor deposition process and wet‐transfer technology, which has a relatively lower cost. 2D material SAs for ultrafast lasers have been studied intensively, making them promising for practical applications. Nevertheless, how the various band structures of 2D materials affect their nonlinear optics is yet to be systematically reviewed. As shown in **Figure** **1**, according to the characteristics of energy band structure, the 2D materials‐based SAs can be classified into three types: traditional 2D semiconductors with parabolic band structure (e.g., BP,^[^ 22, 23 ^]^ TMDs,^[^ ^24^ ^]^ 2D perovskites,^[^ ^25^ ^]^ etc.), Dirac materials (e.g., carbon nanotubes,^[^ ^26^ ^]^ graphene,^[^ ^27^ ^]^ and topological insulators (TIs, including insulating state and Dirac surface state, such as Bi_2_Te_3_, Bi_2_Se_3_, Sb_2_Te_3_, etc.), and flat‐band (FB) materials (e.g., BSCCO,^[^ ^28^ ^]^ include Bi‐2201, Bi‐2212, Bi‐2223, Bi‐2234) with nonparabolic band structure. 2D semiconductors are the most widely studied real SAs. SAs based on 2D BP^[^ ^29^ ^]^ and quasi‐2D perovskite films,^[^ ^30^ ^]^ as direct‐bandgap‐semiconductor SAs, have been proven to have excellent NLO properties, such as high absorption coefficients, high intrinsic electron mobility, and high charge carrier mobility. Research on Dirac materials in recent years has shown that graphene is a kind of SA with good performance^[^ 31, 32, 33, 34, 35 ^]^ due to its properties, such as femtosecond‐class ultrafast electron relaxation time and ultrawide spectral working range. Beyond graphene, many other Dirac materials^[^ ^36^ ^]^ can also be used as 2D SAs, such as germanene^[^ ^37^ ^]^ and silicene,^[^ ^38^ ^]^ which have the same magnitude of intrinsic carrier mobility as graphene. Still, the electron–optical phonon coupling is about 25 times smaller than graphene.^[^ ^39^ ^]^ In addition, the symmetry of the sublattice can be broken in silicene under different external vertical electric field energies, which opens a bandgap at the K point and K′ point. This is impossible for graphene, where two sublattices lie on a plane.^[^ ^40^ ^]^ However, some other 2D materials with unusual electronic structures have been largely unexplored but remained fascinating. The electronic structure of bulk single‐crystal Bi‐2212 has a very flat energy band at the Fermi level, which results in many unusual physical properties, such as a very high superconductivity transition temperature.^[^ ^41^ ^]^ Another study, ref. [28], demonstrated that the 2D Bi‐2212 could be an ideal SA for ultrafast photonics. Although the experimental research on the photonic properties of Dirac and FB materials is still at an early stage, the exotic optical properties of those materials promise new opportunities for ultrafast photonics and optoelectronic applications.

Here, we review the NLO properties of emerging 2D nanomaterials and their applications in ultrafast photonics (i.e., mode‐locked or Q‐switched lasers) and focus on the 2D Dirac materials and FB superconducting nanomaterials with a nonparabolic band structure. We compare and summarize the traditional 2D materials with parabolic band structures (2D semiconductor) and two types of emerging 2D materials with nonparabolic band structures, that is, a graphene‐like kind of material with a Dirac cone band structure and a type of superconducting material with a FB structure, respectively. These materials’ electronic band structures and NLO properties were introduced first. Then the carrier dynamics of related 2D materials were compared, and more importantly, the application of these 2D materials as SAs in ultrafast lasers was reviewed. Finally, we give a summary and outlook. This review will bring new insights into developing emerging 2D photonic materials with nonparabolic bands.

## 2 Electronic Energy Band Structure

**Figure 2:**
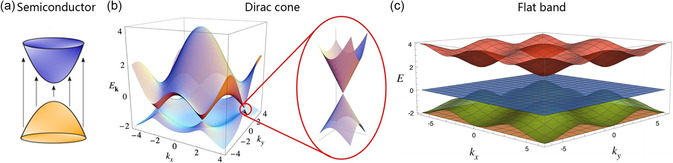
3D schematic diagrams of the band structures. a) Standard semiconductor with parabolic bands. Reproduced with permission.^[^ ^131^ ^]^ Copyright 2018, American Chemical Society. b) Dirac material with Dirac cones. Reproduced with permission.^[^ ^132^ ^]^ Copyright 2009, American Physical Society. c) FB material. Reproduced with permission.^[^ ^133^ ^]^ Copyright 2020, American Physical Society.

The electronic band structure of 2D material determines many characteristics, especially its electronic and optical properties. Band theory is the most basic concept to explain the difference between metals, semiconductors, and insulators. The electronic band structure of crystalline material can be predicted by ab initio calculations or experimentally measured by angle‐resolved photoemission spectroscopy (ARPES). The electronic band structures of metals, semiconductors, and insulators are parabolic, and they are usually distinguished by the energy gap interval. The energy gap of metals is tiny, and electrons can easily gain energy and jump to the conduction band to conduct electricity. In contrast, the energy gap of insulating materials is usually greater than 5 eV. It is difficult for electrons to jump to the conduction band, so they cannot conduct electricity. Generally, the energy gap of semiconductor materials is about 1–3 eV between conductors and insulators. Besides, there are some special electronic band structures, such as Dirac material (i.e., cone band) and FB material (i.e., completely FB). The 3D schematic diagrams of the three types of energy band structures are shown in **Figure** **2**. The schematic diagram of the energy band structure of traditional 2D semiconductor material is shown in Figure 2a. One example is single‐layer TMD with a direct bandgap, high carrier mobility, and good mechanical and optical properties. For its NLO properties, when the material dimension is reduced to 2D, the second‐harmonic generation of the materials (e.g., MoS_2_, WS_2_, WSe_2_) increases significantly.^[^ 42, 43 ^]^

2D materials with Dirac cone or FB are special cases. Dirac cone is characterized by a zero bandgap, as shown in Figure 2b, while FB has a very flat energy band (see Figure 2c). The main feature of 2D Dirac materials is that they show a half‐integer quantum Hall effect^[^ 44, 45 ^]^ and higher carrier mobility.^[^ ^46^ ^]^ Dirac materials have an ultrafast optical response and broadband optical absorption,^[^ 47, 48 ^]^ and they are undoubtedly ideal candidates for ultrafast mode‐locked laser materials SAs. Still, it requires strict synthesis conditions, suitable substrate, and material crystallinity, which is also the reason for the scarcity of Dirac cones. The most significant feature of FB materials is that they can produce dispersionless energy bands. The generation of FBs is due to destructive interference, which leads to macrodegenerate eigenstates in a limited number of unit cells. This kind of macroscopic degeneracy is highly sensitive to disturbances, so even a slight irritation will increase the degeneracy and cause various unusual physical phenomena.^[^ ^49^ ^]^ At present, FBs are being studied in various fields of condensed matter physics and have been experimentally realized in the 2D system. Many theoretical and experimental studies have been implemented on FBs in photonic crystal waveguide networks and optical waveguide arrays at room temperature.^[^ 49, 50, 51, 52, 53 ^]^ Compared with the linear Dirac band carrying massless quasiparticles, the dispersionless FB makes the electrons overweight due to its localization.^[^ 54, 55, 56 ^]^ The Dirac band sharply contrasts with FB, which has completely different physical properties. The unique electronic structures afford FBs many effects, such as the high‐temperature fractional quantum Hall effect,^[^ ^57^ ^]^ high‐temperature superconductivity,^[^ ^58^ ^]^ and so on. FB materials have brought excellent prospects in the field in terms of lattice‐dependent optical transition^[^ ^59^ ^]^ and local nonlinear dynamics,^[^ ^60^ ^]^ which endow them with great promise for nonlinear optics and ultrafast photonics.

**Figure 3:**
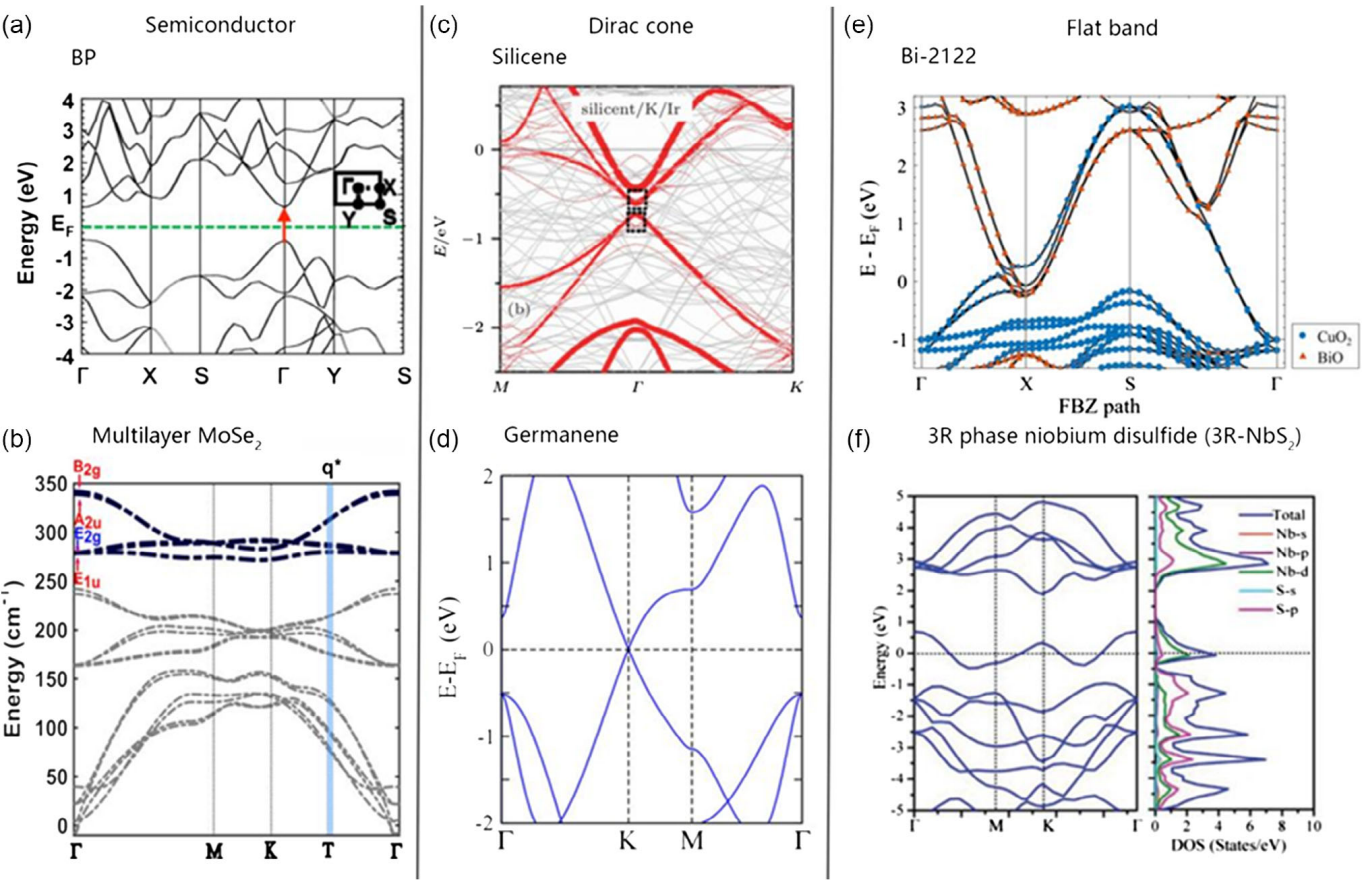
a–f) Ab initio calculation of band structure: a) BP; b) multilayer MoSe_2_; c) silicene; d) germanene; e) Bi‐2212; and f) 3R‐NbS_2_. a) Reproduced with permission.^[^ ^8^ ^]^ Copyright 2014, American Chemical Society. b) Reproduced with permission.^[^ ^134^ ^]^ Copyright 2022, American Physical Society. c) Reproduced with permission.^[^ ^74^ ^]^ Copyright 2015, IOP Publishing. d) Reproduced with permission.^[^ ^135^ ^]^ Copyright 2013, American Institute of Physics. e) Reproduced with permission.^[^ ^136^ ^]^ Copyright 2021, IOP Publishing. f) Reproduced with permission.^[^ ^113^ ^]^ Copyright 2019, Wiley‐VCH.

The typical electronic band structures of the three types of materials based on ab initio calculations are shown in **Figure** **3**. The first type of 2D semiconductor material, for instance, BP and multilayer MoSe_2_, has a small bandgap located between the zero‐bandgap in graphene and the large bandgap in TMDs (see Figure 3a,b), attracting extensive research interest in this type of material.^[^ ^61^ ^]^ BP is the intrinsic counterpart of phosphorene and the most stable phosphorus allotrope. Its layered structure combines van der Waals weak interlayer forces, similar to graphite. Phosphorene has the characteristics of a typical 2D material, such as layer‐count‐dependent electronic characteristics. The direct bandgap of phosphorene ranges from 1.73 eV for a single layer, 1.15 eV for a double layer, 0.83 eV for three layers, to 0.35 eV for bulk.^[^ ^62^ ^]^ Based on the ab initio calculation, monolayer free‐standing phosphorene has a direct bandgap of 1.0 eV at the Γ point (see Figure 3a), which is significantly larger than the bulk value (0.31 eV). The in‐plane puckered structure of BP rather than a center symmetry leads to anisotropic conductivity and photoconductivity. Thus, light absorption and photoluminescence of BP have a high degree of anisotropy.^[^ ^29^ ^]^ As a novel 2D nanomaterial, BP has been intensively studied in NLO devices due to its high carrier mobility, tunable direct‐bandgap characteristics, and unique in‐plane anisotropic structure. However, BP is easily oxidized and degraded after encountering water vapor and oxygen in the air; it is necessary to use surface passivation or functionalization or a protective layer if the practical application is to be developed.

Graphene is a widely studied typical Dirac material with a Dirac cone band structure.^[^ 44, 45 ^]^ Inspired by the high performance of graphene in applications, researchers began to explore the elements of the IVA main group with carbon. Besides graphene, silicene, and germanene,^[^ ^63^ ^]^ several graphenes (sp–sp^2^ carbon allomorphs)^[^ 64, 65 ^]^ and other systems^[^ 66, 67 ^]^ were predicted to be Dirac materials in early studies; it has been gradually proven to retain Dirac properties in recent studies.^[^ 37, 68 ^]^ The Dirac characteristics and band structure of silicene and germanene have recently been reported^[^ ^40^ ^]^ (Figure 3c,d). Silicene and germanene are similar to graphene in many respects and have the same hexagonal structure as graphene. But unlike graphene's flat hexagonal crystal structure, they have a low‐bending honeycomb structure. Since silicon and germanium have atomic numbers than carbon, they exhibit stronger spin–orbit coupling than carbon. The difference in material properties caused by the difference in structure dramatically influences the electronic properties and brings new physical properties different from graphene. Slight buckling will increase the spin–orbit coupling by an order of magnitude.^[^ ^69^ ^]^ The carbon atoms that make up graphene are *sp* ^2^ hybridized, while the atoms that form silicene/germanene are more inclined to *sp* ^3^ hybridization, a 3D covalent bond configuration. Therefore, a layered structure similar to graphite in nature would not be observed in silicene or germanene, and it is impossible to obtain a monoatomic layer simply by mechanical exfoliation. In this regard, silicene or germanene must be artificially manufactured and grown on specific substrates. However, the interaction with the substrate cannot be ignored.

The controllable preparation of germanene has become a research hotspot in recent years. In 2013, Bianco et al.^[^ ^70^ ^]^ reported on the preparation and exfoliation of germanane (GeH) for the first time, which opened the research on synthesizing germanium and germanium‐related materials. The support of metal substrates can stabilize 2D germanene;^[^ 71, 72, 73, 74, 75, 76, 77, 78 ^]^ the rich superstructure is formed due to different kinds of germanene–substrate interaction. However, by interacting with metal substrates such as Ag(111),^[^ ^73^ ^]^ Pt(111),^[^ ^71^ ^]^ and Au(111),^[^ ^75^ ^]^ despite the formation of a 2D germanene superstructure with a low‐buckling honeycomb atomic arrangement, the superstructure induced by the substrate will destroy the out‐of‐plane symmetry and cause the loss of the Dirac fermion properties.^[^ ^79^ ^]^ The retention of Dirac properties of germanene has recently been confirmed. In 2017, Qin et al.^[^ ^72^ ^]^ first reported the growth of island‐shaped double‐layer germanene on Cu(111), proving a nearly linear energy dispersion near the Fermi energy. In 2018, Zhuang et al.^[^ ^80^ ^]^ successfully prepared monolayer germanene on the surface of semiconductor Ge(111), which greatly reduces the hybridization effect induced by the substrate and can retain and solve the Dirac characteristics. Mu et al.^[^ ^37^ ^]^ prepared a germanene sample by heating a Ge wafer to evaporate and deposit Ge atoms on the Ag(111) surface.

Germanene has a Dirac cone structure near the Γ point in the Brillouin zone. The linear energy dispersion is similar to that of graphene, indicating the existence of Dirac fermions. In linear dispersion, the Fermi velocity of *ν*_F_ = 1.03(±0.2) × 10^6^ ms^−1^ is obtained by calculation, which is close to that by the ab initio calculation^[^ ^63^ ^]^ and in the Ge slice/Au(111)^[^ ^76^ ^]^ system. As reported in previous studies,^[^ 81, 82 ^]^ the bandgap of Ge(111) is ≈0.65 eV, so the Dirac feature should be related to the top germanium layer. It has been verified that the Fermi level is at ≈0.5 eV by extracting the constant energy surface mapping. A high modulation depth of 13.9% has been realized using Ge nanosheets as SA, with low saturable intensity (2.266 GW cm^−2^), low nonsaturable loss (11.90%), and relatively fast relaxation (901 fs).

As a FB material, BSCCO (Type II superconductor) is a family of high‐temperature superconductors with a general chemical formula: Bi_2_Sr_2_Ca_*n*−1_Cu_*n*_O_2*n*+4+δ_, where *n* = 1–3. Bi‐2212 is one of the most studied compounds, and its chemical formula is Bi_2_Sr_2_CaCu_2_O_8+δ_, whereas *n* = 2. Among them, pseudogap,^[^ 83, 84 ^]^ infrared Hall angle,^[^ ^85^ ^]^ and superconductivity^[^ ^86^ ^]^ of bulk single‐crystal Bi‐2212 have attracted widespread attention. The band structure of Bi_2_Sr_2_CaCu_2_0_8+δ_ near the Fermi energy by ab initio calculation along the Γ–X–S–Γ path of the first Brillouin zone (FBZ) consists of the conclusion that there is an FB near *E*_F_ in the p‐type cuprate Bi‐2212 by theoretical calculations from the previous study^[^ ^41^ ^]^ (see Figure 3e). The energy band structure of 2D van der Waals metal 3R phase niobium disulfide (3R‐NbS_2_) is shown in Figure 3f. Bi‐2212 and 3R‐NbS_2_ are FB materials with superconductivity. Bi‐2212 crystal has a 2D layered lattice similar to other high‐temperature superconductors, in which two Bi_2_O_2_ layers separate the CuO_2_ plane. Similar to other 2D materials, the few‐layer Bi‐2212 nanosheets can be produced by a micromechanical exfoliation process. The investigation of the single‐layer Bi‐2212 shows that its influence on the superconducting phase, charge order, pseudo‐gap, and Mott state under different doping concentrations is relatively small, the same as the bulk counterpart.

**Figure 4:**
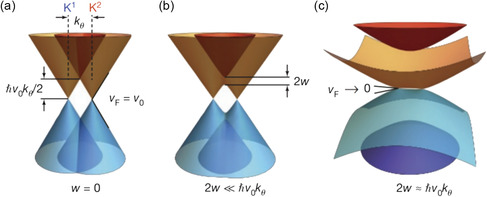
a–c) Illustration of the effect of interlayer hybridization for *w* = 0 (a), 2*w* <<* ħv*_0_ *k*_*θ*_ (b) and 2*w* ≈* ħv*_0_ *k*_*θ*_ (c). *v*_*0*_ = 10^6^ m · s^−1^ is the Fermi velocity of graphene. a–c) Reproduced with permission.^[^ ^88^ ^]^ Copyright 2018, Springer Nature.

Several research results are worth considering about the Dirac cone and FB band structure. Shen et al.^[^ ^87^ ^]^ used ultracold atoms fixed in an optical lattice to form a line‐centered‐square lattice. By adjusting the laser intensity, the behavior of Dirac fermions can be transformed from mass to massless. With the appearance of a single Dirac cone, the FB contacts the Dirac point and the vanishing Berry's phase in the energy spectrum. Cao et al.^[^ ^88^ ^]^ experimentally demonstrated that when the torsion angle of twisted bilayer graphene (TBG) is close to the magic angle (MA) predicted by theory, interlayer hybridization induces an almost flat low‐energy band (**Figure** **4**). To zeroth order, the low‐energy band structure of TBG can be considered as two sets of monolayer‐graphene Dirac cones rotated around the Γ point in the Brillouin zone by a twist angle *θ*. The difference between the two K (or K′) wave vectors produces the mini‐Brillouin region (shown as a small hexagon), which is caused by the reciprocal lattice of the Moiré super lattice. Figure 4 implies that the twist angle leads to the contrast between the different hybrid energies 2w and the intrinsic energies. When the hybrid energy 2w is equal to or greater than it, the low energy of the hybrid state is pushed to and beyond the zero energy. Figure 4 depicts that twist angles lead to different hybridization energies 2w versus the intrinsic energy $\hbar \nu_{0}k_{\theta }$. While the hybridization energy 2w is comparable to or larger than $\hbar \nu_{0}k_{\theta }$, the lower of the hybridized states is pushed and crosses zero energy. It is proved that a FB may exist in the theoretical prediction of TBG,^[^ ^89^ ^]^ and the level crossing of two Dirac cones produces a FB at the junction, thus making TBG an accurate tunable, pure carbon‐based, 2D superconductor. This is also the source of the superconductivity in MA‐TBG.^[^ 90, 91 ^]^ From Dirac cone to FB, it largely predicts the future development direction of 2D materials. The proof of MA shows the potential of material properties and uncovers many unexplored areas in the materials land. Finding the MA of other 2D materials and figuring out the characteristics of the materials will be new challenges.

Using ultrashort pulses to excite 2D materials will lead to special nonlinear responses and coherent dynamics changes in 2D materials. For example, Floque engineering offers theoretical possibilities for the optical manipulation of the topological quantum states of Dirac materials.^[^ 92, 93, 94 ^]^ The quantum states of the Dirac material can be dressed using circularly polarized light to form the hybridization at the Dirac intersection and opening its bandgap. Thus, the abnormal Hall effect of the Dirac cone is induced. The redox‐exfoliated TMDs have attracted much attention as photonic devices due to their linear and NLO properties, including saturable absorption and photoluminescence.^[^ 95, 96 ^]^ There occurs nonlinear interaction between intense pulse on a much shorter time scale and the electron band structure in a more complex way. The NLO effect, carrier‐wave Rabi flopping, can be observed in GaAs.^[^ ^97^ ^]^

## 3 Ultrafast Dynamics

**Figure 5:**
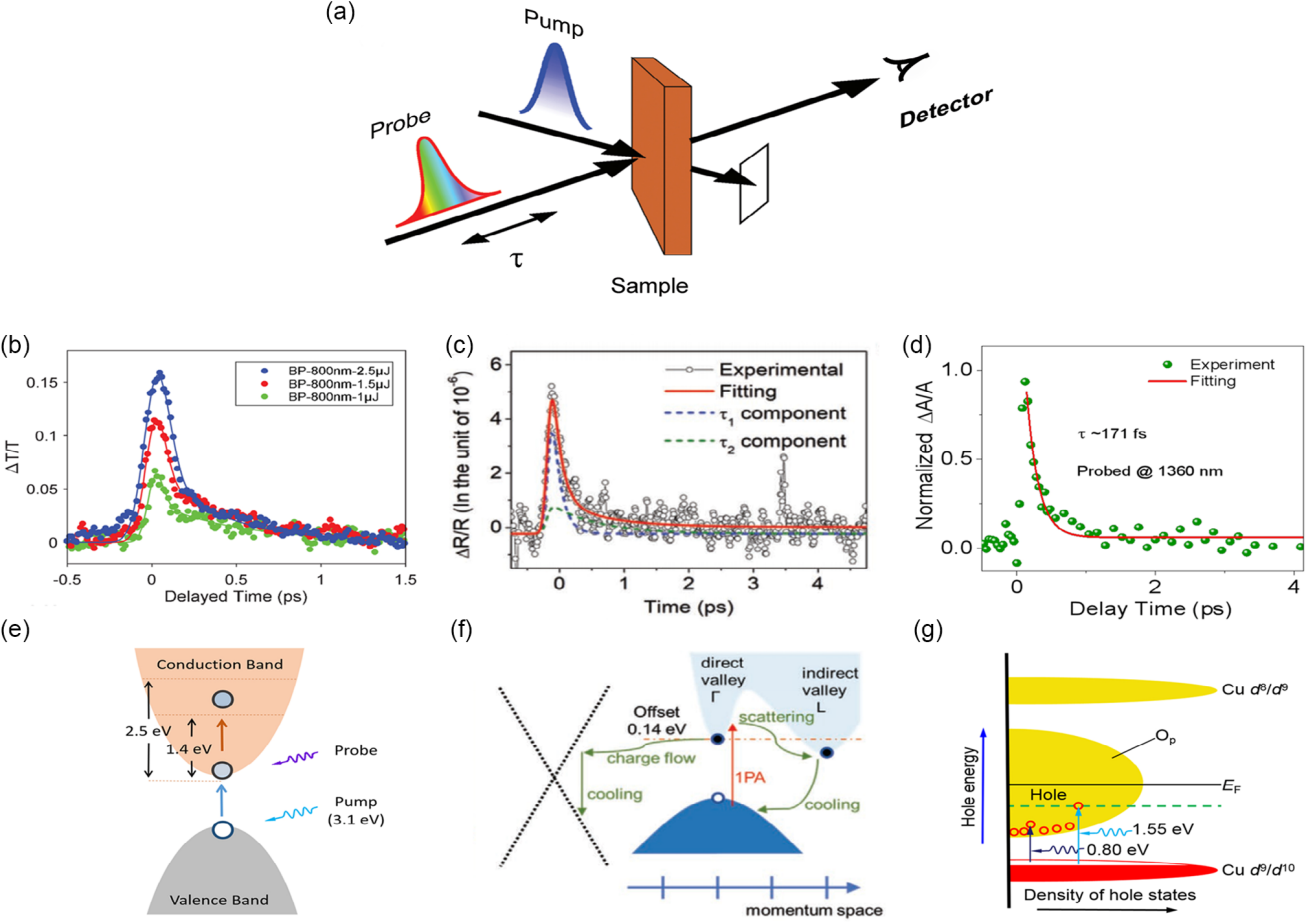
a–c) Pump–probe results. a) Schematic of pump–probe process. b) BP; c) Ge nanosheets; d) Bi‐2212 flakes. b) Reproduced with permission.^[^ ^22^ ^]^ Copyright 2016, American Chemical Society. c) Reproduced with permission.^[^ ^37^ ^]^ Copyright 2021, Wiley‐VCH. d) Reproduced with permission.^[^ ^28^ ^]^ Copyright 2021, American Chemical Society. e–g) Energy diagrams of the optical process: e) BP; f) Ge nanosheets; g) Bi‐2212 flakes. e) Reproduced with permission.^[^ ^137^ ^]^ Copyright 2017, Optica Publishing Group. f) Reproduced with permission.^[^ ^37^ ^]^ Copyright 2021, Wiley‐VCH. g) Reproduced with permission.^[^ ^28^ ^]^ Copyright 2021, American Chemical Society.

An ultrafast carrier recovery time is necessary for materials in ultrafast photonics. The pump‐probe experiment can characterize the carrier dynamics of the materials. The schematic of the pump‐probe process is shown in **Figure** **5**a. A first energetic pump pulse is absorbed by the sample generating an excited state, while a second delayed and weaker pulse tracks the pump‐induced changes in the optical response. The probe can be either an attenuated replica of the pump pulse, obtained by a beam splitter. The relaxation of carriers can be revealed by analyzing the differential transmission trajectory as a function of delay time. This section summarizes the recent experimental studies on the ultrafast dynamics of three material types: traditional 2D semiconductors with parabolic band structure, Dirac materials, and FB materials with a nonparabolic band structure. The effects of ultrafast dynamics on the carrier recovery time in these three types of materials are discussed.

MoS_2_ and BP are typical semiconductors with parabolic band structures. For example, single‐layer MoS_2_ is a direct‐bandgap semiconductor with an energy gap of ≈1.9 eV, while multilayer MoS_2_ is an indirect‐bandgap semiconductor with an energy gap of ≈1.2 eV.^[^ 98, 99 ^]^ For photons with an energy of 1.55 eV (*λ* = 800 nm), the monolayer can only be excited by absorbing two photons, with TPA, and multilayer MoS_2_ can be excited by one photon, with saturable absorption (SA). BP shows an obvious two‐component response to the pump–probe trace: two relaxed parts *τ*_1_ and *τ*_2_. Under the 800 nm pulse, the average fast component *τ*_1_ of BP is 16 fs, in a coherent state, and is lower than the pulse duration of ≈100 fs. The slow component *τ*_2_ is 360 fs, and the relaxation is in a nonthermal state due to carrier‐phonon scattering. The maximum value of ΔT/T increases roughly linearly with the increase of the pump pulse energy. *τ*_2_ at 800 nm indicates the existence of ultrafast carrier relaxation in BP on the femtosecond scale (Figure 5b). The pump wavelength is 400 nm (photon energy = 3.1 eV), which excites the carriers of BP, and the continuous broadband photon‐induced absorption (PIA) signal from 470 to 850 nm infers that the conduction band of the continuous band structure of BP is 1.4–2.5 eV (Figure 5e).

In the previous work, Mu et al. demonstrated that germanene is a Dirac material.^[^ ^37^ ^]^ To study the carrier dynamics of germanene, the change of the probe pulse transient reflection (ΔR) was recorded to resolve the cooling process of hot carriers. The transient reflectance spectra of germanene with a thickness of 8–12 nm (corresponding to 20–36 L) were drawn by normalizing the probe reflectance (Δ*R*/*R*) (Figure 5c). The fast component *τ*_1_ represents the carrier scattering of pump and probe pulses covering the autocorrelation (AC) range. The slow component *τ*_2_ represents the electron–phonon scattering and the diffusion of carriers from the bulk state to the Dirac state. D_1_ and D_2_ correspond to the amplitude of *τ*_1_ and *τ*_2_. *τ*_1_ and *τ*_2_ of the 20–36 L germanene sample are 128 fs and 0.73 ps, respectively, while the amplitude ratio of D_1_/D_2_ is as high as 8.54, reflecting the dominance of the *τ*_1_ process in the sample. Compared with the upper limit of bulk Ge (230 fs),^[^ ^100^ ^]^ the fast relaxation time of Ge quantum well is ≈185 fs,^[^ ^101^ ^]^ and the results show a faster relaxation process. Electrons are excited from the valence band to the conduction band by absorbing the energy of the pump photons. They can be further excited by the probe photons to a higher conduction band state, resulting in additional absorption by the probe laser.^[^ ^23^ ^]^ The energy diagram of the optical process of germanene reveals the process of electronic transition (Figure 5f). The main process of germanene electronic transition is the optical transition between single‐photon bands.

The carrier dynamics of FB superconducting materials at different temperatures in the past few years have been extensively studied.^[^ 102, 103, 104, 105 ^]^ The carrier dynamics of plasmon resonance in 2D ≈125 nm‐thick Bi‐2212 nanosheets at room temperature through ultrafast pump–probe experiments have been studied. The transient absorption spectrum in the near‐infrared region was pumped at 800 nm and detected at a wavelength of 1360 nm (Figure 5d). The carrier relaxation time *τ* of the 2D Bi‐2212 is ≈170 fs, which is comparable to 150 fs of 1–4 L graphene^[^ ^106^ ^]^ and much faster than some widely studied materials, such as TMDs,^[^ ^107^ ^]^ MXenes,^[^ ^108^ ^]^ etc. Like metals, the bolometric effect is generally considered to affect the transient absorption response of Bi‐2212, that is, the thermal process leads to the attenuation of the transient absorption response, which is independent of the recombination of nonequilibrium carriers.^[^ ^102^ ^]^ Electron–plasmon interaction plays a decisive role in the energy‐dependent relaxation time at the beginning. Specifically, a thermal interaction between the hot carriers will occur after a laser pulse excites. In this thermal process, electron–electron collisions occur, resulting in the avalanche multiplication of quasiparticles.^[^ ^103^ ^]^ After that, quasiparticles’ energy is lost to the lattice when they emit phonons. In addition, part of the energy of the incident pump pulse excites the d^9^/d^10^‐like state of the hole to the copper oxygen p‐like state and the remaining energy heats the charge carriers in the metallic phase. It will cause the filled state (oxygen p‐like states) to be above and the open state (d^9^/d^10^‐like states) to be below (Figure 5g). The ultrafast increase in the normalized difference of absorption Δ*A*/*A* indicates that the pump opens more states of absorption.^[^ ^109^ ^]^ The absorptance drops to a slow attenuation background associated with carrier‐phonon coupling.

## 4 NLO Properties

Due to dimensional effects, 2D materials have a stronger NLO response than intrinsic materials. The known nonlinear effects in 2D materials include the Kerr effect and saturable absorption. The former is generally measured using the closed‐aperture Z‐scan technique, which can give the real part of the third‐order magnetic susceptibility *χ* ^(3)^ and the nonlinear exponential parameter n_2_. The latter measured by the open‐aperture Z‐scan technique is a kind of nonlinear absorption related to the imaginary part of *χ* ^(3)^. By fitting the z‐scan curve, one can get the value of linear absorption coefficient α_0_ and nonlinear absorption coefficient *β*. Therefore, Im^(3)^ and the figure of merit (FOM) can be calculated. Using a mode‐locked pulse of a specific center wavelength as the excitation source and measuring the transmittance of the material with the incident power density, one may have the saturation absorption characteristics of the material at a specific wavelength. After fitting,^[^ ^110^ ^]^ the transmittance, unsaturable absorption coefficient, saturable absorption coefficient, saturated intensity *I*_sat_, and modulation depth of the SA can be obtained. The higher the modulation depth value, the stronger the pulse intensity modulation ability of the material. Here, we have compared the nonlinear characteristics of traditional 2D semiconductors with emerging Dirac and FB materials by summarizing experiments results from pump‐probe, Z‐scan, and nonlinear absorption measurements.

**Figure 6:**
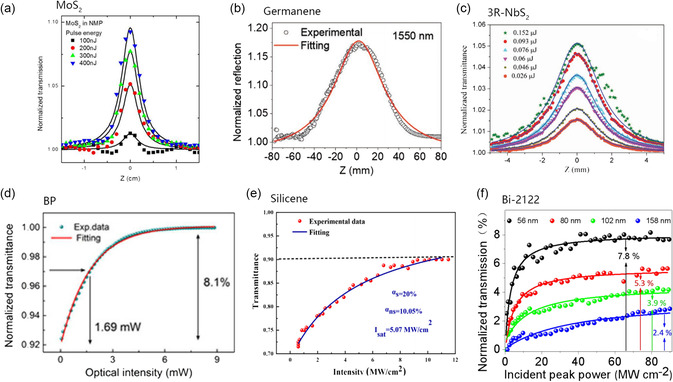
a) Open‐aperture Z‐scans of the MoS2 dispersion under the excitation of 100 fs pulses at 800 nm. Reproduced with permission.^[^ ^24^ ^]^ Copyright 2013, American Chemical Society. b) Open‐aperture Z‐scan measurements for NLO properties of germanene nanosheets with 8–12 nm thickness. Reproduced with permission.^[^ ^37^ ^]^ Copyright 2021, Wiley‐VCH. c) Open‐aperture Z‐scan characterizations of 2D 3R‐NbS_2_ nanosheets under picosecond pulse excitation at 1064 nm with different incident pulse fluences. Reproduced with permission.^[^ ^113^ ^]^ Copyright 2019, Wiley‐VCH. d) Saturable absorption property of the BP‐SA device. Reproduced under the terms of the CC‐BY Creative Commons Attribution 4.0 International license (https://creativecommons.org/licenses/by/4.0).^[^ ^114^ ^]^ Copyright 2017, The Authors, published by Springer Nature. e) Nonlinear absorption property of silicene nanosheets. Reproduced with permission.^[^ ^38^ ^]^ Copyright 2020, Elsevier Ltd. f) Nonlinear transmission of Bi‐2212 flakes with different thicknesses. Reproduced with permission.^[^ ^28^ ^]^ Copyright 2021, American Chemical Society.

In the Z‐scan curves of three types of 2D materials with different energy band structures, all curves have sharp and narrow peaks at the focus position, indicating the saturation absorption characteristics of the materials (see **Figure** **6**a–c). The NLO parameters of the three materials types are presented in **Table** **1**. Wang et al.^[^ ^24^ ^]^ used the excitation of 100 fs mode‐locked pulse at 800 nm to study the ultrafast NLO behavior of dispersion of molybdenum disulfide nanosheets, as shown in Figure 6a. When the excitation energy is increased to 100 nJ per pulse, the NLO response occurs in molybdenum disulfide, for which the corresponding focus intensity is 65.8 GW cm^−2^, and the flux is 6.6 mJ cm^−2^. When the excitation energy is greater than 200 nJ per pulse (≈131.6 GW cm^−2^), the MoS_2_ dispersion exhibits a significant SA response, indicating that molybdenum disulfide nanosheets can effectively suppress low‐intensity light but allow high‐intensity light to pass through. The absorption saturation of the fs pulse in the NIR region suggests that the molybdenum disulfide nanosheets can be used as an ultrafast nonlinear SA and a passively mode‐locked element for ultrashort pulse lasers. The open aperture z‐scan result of the germanene sample was measured at 1550 nm, as presented in Figure 6b. It exhibits a typical saturable absorption response consistent with the pump‐probe results. The third‐order NLO polarizability Im*χ* ^(3)^ of 20‐36 L Ge at 1550 nm is −(2.72 ± 0.53) × 10^−8^ esu, which is almost 30 times that of graphene and 200 times that of MoS_2_,^[^ ^111^ ^]^ at least twice that of WS_2_.^[^ ^112^ ^]^ Sun et al.^[^ ^113^ ^]^ used a 1064 nm Yb‐doped fiber laser with a pulse width of 15 ps to perform z‐scan measurements on 2D 3R‐NbS_2_, as depicted in Figure 6c. When the maximum incident flux is 402 μJ cm^−2^, no reverse saturable absorption caused by TPA, nonlinear scattering, or a combination can be observed. The average values of the measured nonlinear absorption coefficient *β*_eff_ and Im*χ* ^(3)^ are −2.5 × 10^6^ cm GW^−1^ and −2.4 × 10^−6^ esu, respectively, which remain almost constant at 1064 nm, indicating that the single‐photon saturable absorption process dominates 2D 3R‐NbS_2_ nonlinear absorption process.

**Table 1:** Nonlinear optical parameters of three types of 2D materials

Type	Material	*λ* [nm]	*α*_0_ [cm^−1^]	*β* [cm GW^−1^]	Im*χ* ^(3)^ [esu]	FOM [esu cm]	*I*_sat_ [GW cm^−2^]	References
Semiconductor	MoS_2_	800	1.3 × 10^4^	−136.13	−0.3 × 10^−9^	4.6 × 10^−14^	347.6	[111]
	WS_2_	800	7.22 × 10^5^	−397 ± 40	−(1.78 ± 0.2) × 10^−9^	(2.47 ± 0.23) × 10^−15^	–	[112]
	BP	800	8.7	−0.0138	−0.785 × 10^−14^	–	459	[22]
Dirac	Graphene	800	4.6 × 10^4^	−961.57	−2.4 × 10^−9^	3.1 × 10^−14^	166.1	[111]
	Gemanene	800	(1.24 ± 0.25) × 10^5^	−(1.53 ± 0.31) × 10^4^	−(6.50 ± 1.31) × 10^−8^	(5.26 ± 0.02) × 10^−13^	16.4 ± 0.2	[37]
		1550	(0.94 ± 0.19) × 10^5^	−(3.12 ± 0.61) × 10^3^	−(2.72 ± 0.53) × 10^−8^	(2.88 ± 0.04) × 10^−13^	1.04 ± 0.08	
	Silicene	532	7.5	4.8	3.7 × 10^−5^	6.9 × 10^−16^	6.9	[138]
		1064	6.5	3	4.6 × 10^−5^	9.9 × 10^−16^	54.1	
Flat Band	3R‐NbS_2_	1064	3.3 × 10^5^	−2.5 × 10^6^	−2.4 × 10^−6^	7 × 10^−12^	33 MW cm^−2^	[113]

The transmittance curves of the three types of materials are depicted in Figure 6d–f. The NLO properties of the three types of materials with different thicknesses are summarized in **Table** **2**. Du et al.^[^ ^114^ ^]^ used a homemade 1560 nm‐wavelength ultrashort fiber laser light source as the pump light source and obtained the nonlinear transmission curve of ≈2.6 nm BP‐SA (Figure 6d). It can be concluded that the saturated average power and normalized modulation depth of the device are 1.69 mW and 8.1 %, respectively. The fitting value of the modulation depth of silicene‐SA with an average thickness of ≈6.2 nm at a wavelength of 1550 nm is 20 %, and the saturation intensity is 5.07 MW cm^−2^, indicating that the SA has strong saturation absorption characteristics (Figure 6e). In our previous work,^[^ 28, 115 ^]^ we obtained the nonlinear transmission curve of ≈125 nm Bi‐2212 by measuring the function of the incident laser power at 1550 nm (Figure 6f). It is found that the transmittance of all 2D Bi‐2212 nanosheets increases significantly at a specific incident laser intensity and then reaches saturation at a higher intensity power. As the thickness increases from 56 nm to 158 nm, the normalized nonlinear transmittance decreases from 7.8% to 2.4%. As the thickness increases, the scattering of the multilayer Bi‐2212 is enhanced, increasing unsaturable loss, which leads to a decrease in the modulation depth. The final modulation depth of the Bi‐2212 sample is 2.9%, and the saturation intensity is about 28 MW cm^−2^, which is greater than the few‐layer graphene but less than MXene, bismuth telluride, and molybdenum disulfide, indicating that Bi‐2212 may be an ideal SA candidate for ultrafast photonics.

**Table 2:** The NLO performance of different 2D samples

Type	Material	Modulation depth	Saturable intensity [MW cm^−2^]	Non‐saturable loss	References
Semiconductor	15 L BP	8.10%	6.55	–	[139]
	25 L BP	18.55%	10.74	–	
	≈280 nm Perovskite	27.80%	0.93	–	[119]
Dirac	10–18 L Gemanene	13.90%	2.266 GW cm^−2^	11.90%	[37]
	20–36 L Gemanene	9.02%	3.41 GW cm^−2^	25.47%	
	2–4 L Graphene	66.50%	0.71	33.50%	[140]
	9–11 L Graphene	6.20%	0.61	94.50%	
	≈20 L Silicene	20%	5.07	10.05%	[38]
FB	≈125 nm Bi‐2212	2.90%	28	–	[28]
	≈6 L 3R‐NbS_2_	10.00%	33	–	[113]

## 5 Applications in Mode‐Locked lasers

**Figure 7:**
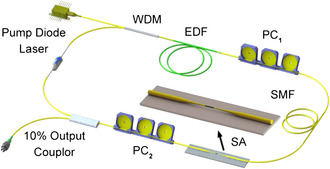
Representative schematic fiber laser cavity. WDM: wavelength‐division multiplexer, EDF: erbium‐doped fiber, PC: polarization controller, SA: saturable absorber, and SMF: single‐mode fiber. Reproduced under the terms of the CC‐BY Creative Commons Attribution 4.0 International license (https://creativecommons.org/licenses/by/4.0).^[^ ^114^ ^]^ Copyright 2017, The Authors, published by Springer Nature.

SA devices used in passively mode‐locked lasers are generally divided into real SAs and artificial SAs. Real SAs include SESAMs based on the basic structure of mirrors and combined semiconductor SAs and 2D nanomaterials with optical characteristics such as slight unsaturated loss, light intensity‐dependent nonlinear absorption, large modulation depth, etc. Artificial SAs generally include three technical approaches, such as nonlinear fiber loop mirror (NOLM), nonlinear magnifying loop mirror (NALM), and nonlinear polarization rotation (NPR). Artificial SA devices use the nonlinear refractive index or birefringence characteristics to induce the intensity‐dependent nonlinear absorption, simulating the response of real SAs. Although the mode‐locking technologies based on real SAs and artificial SAs have different approaches, they are physically equivalent to the saturation absorption effect. Compared with the active method, the passively mode‐locked laser using real SA has significant advantages such as self‐starting operation, low cost, high stability, and no maintenance. Typical fiber laser consists of single‐mode all‐fiber integrated components for an alignment‐free and compact system, as shown in **Figure** **7**. The fiber amplifier consists of single‐mode Er‐doped fiber (EDF), copumped by a 980 nm pump laser diode. The pump light is coupled into the cavity through a reflection‐type 980/1550 nm wavelength division multiplexer (WDM). In addition to the fiber amplifier, the cavity includes a polarization‐independent optical isolator(PI‐ISO) to ensure unidirectional propagation and polarization controllers (PC1 and PC2) to enable a thorough and continuous adjustment of the net cavity birefringence.^[^ ^114^ ^]^ Based on the reliable saturable absorption capacity of 2D semiconductors, the IV main group graphene‐like Dirac materials, and FB superconductors, the application of the three types of materials in mode‐locked lasers is expected.

### 5.1 2D Semiconductor‐Based SAs

SA mode‐locked lasers based on 2D semiconductor materials are the most widely studied real SAs‐based lasers.^[^ ^116^ ^]^ At present, BP, TMDs, and perovskite have received widespread attention and are representative examples.

**Figure 8:**
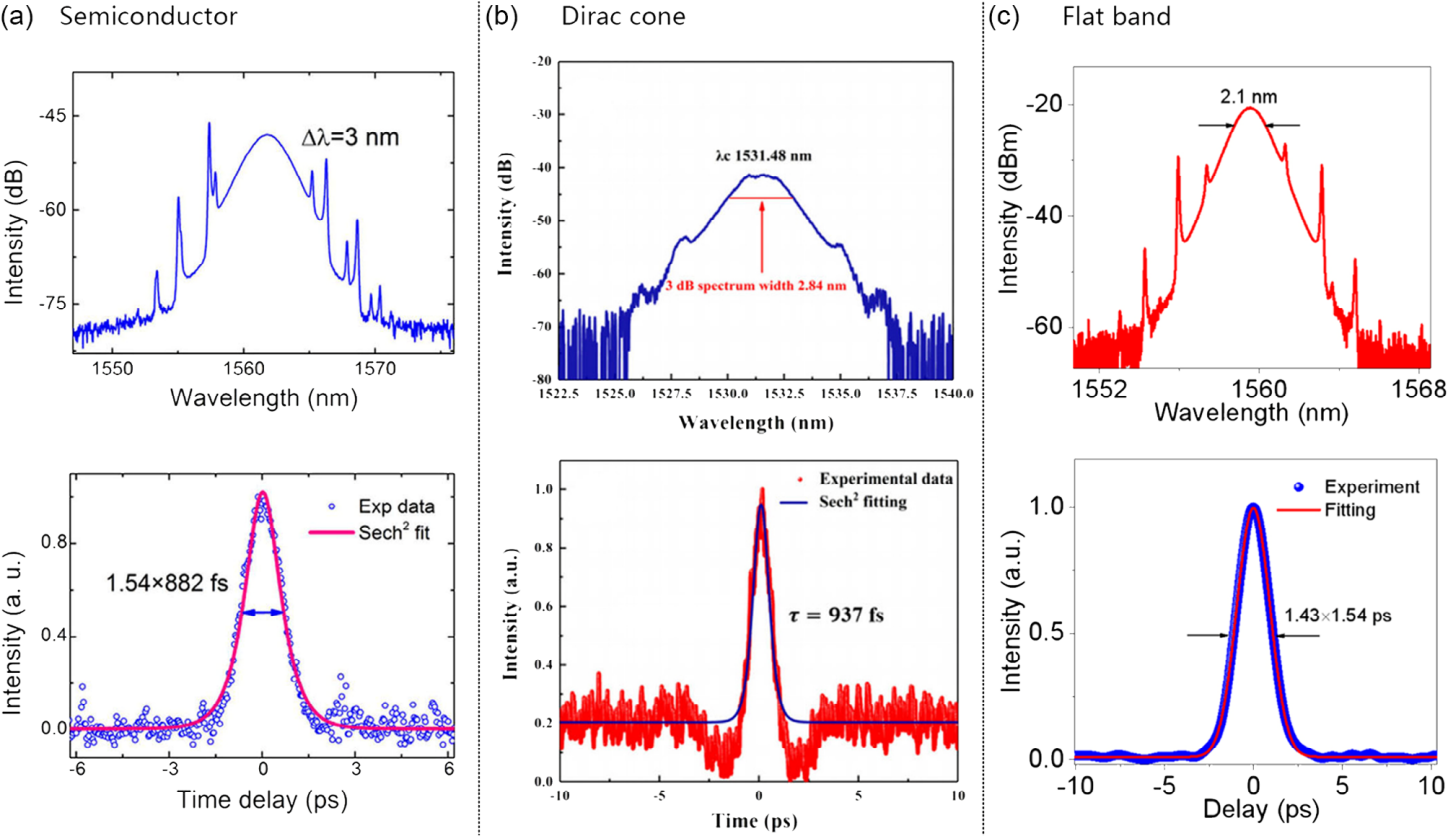
a–c) Typical characteristics of pulses output from the mode‐locked fiber laser. a) BP; B) silicene; c) 2D BSCCO flakes. a) Reproduced under the terms of the CC‐BY Creative Commons Attribution 4.0 International license (https://creativecommons.org/licenses/by/4.0).^[^ ^114^ ^]^ Copyright 2017, The Authors, published by Springer Nature. b) Reproduced with permission.^[^ ^38^ ^]^ Copyright 2020, Elsevier Ltd. c) Reproduced with permission.^[^ ^28^ ^]^ Copyright 2021, American Chemical Society.

Du et al.^[^ ^114^ ^]^ showed that the BP‐SA‐based erbium‐doped laser is self‐starting and mode locked at a fundamental repetition frequency of 5.47 MHz, with single‐pulse energy of 24.7 pJ. The spectrum is centered at 1561.7 nm, and the full width at half maximum (FWHM) is 3 nm, as shown in **Figure** **8**a. Using an intensity autocorrelator to measure the corresponding pulse duration after deconvolution, Sech^[^ ^112^ ^]^ fits the pulse shape very well and gives a pulse width of 882 fs. The time‐bandwidth product (TBP) is calculated as 0.325, close to the Fourier transform limit of the soliton pulse. The fundamental frequency has a high signal‐to‐noise ratio (SNR) of ≈67 dB, which indicates that the mode‐locking operation performance is stable. The laser spectrum was recorded every 20 min within 2 h, and the center wavelength and spectral bandwidth did not change significantly, indicating that the mode‐locking operation has good operational stability.

The unique properties of 2D TMDs, such as saturable absorption, bandwidth adjustability, and variable electron, ensure the function as a pulsed laser in a broad band from visible to NIR.^[^ ^117^ ^]^ Koo et al.^[^ ^118^ ^]^ fabricated a defective WTe_2_‐SA to achieve a 1556 nm femtosecond EDF laser at a repetition rate of 13.98 MHz. The pulse duration was 770 fs, and the SNR was 67 dB. The study proves that the structural defects of TMDs will not change the saturated absorption capacity.

Jiang et al.^[^ ^119^ ^]^ added 2D perovskite‐SA to a ring cavity fiber laser to obtain 661 fs pulses. When the pump power exceeds the threshold of about 70 mW, the single‐pulse mode‐locking operation will start automatically. When the pump power is 120 mW, there is a typical mode locking, the center wavelength of the spectrum is 1555 nm, and the 3 dB bandwidth is 5 nm. The pulse repetition frequency is 13.15 MHz, and the calculated TBP is 0.41. The SNR can reach 61 dB, which verifies the low‐amplitude fluctuation, single‐pulse state, and high stability of the output pulse sequence.

### 5.2 Dirac Materials‐Based SAs

Guowei Liu et al.^[^ ^38^ ^]^ incorporated the microfiber‐based silicon nanosheet SA (Si‐SA) into an EDF laser for the first time. The thickness of silicene nanosheet is ≈6.2 nm, which is about 20 layers. By measuring the transmissivity of the Si‐SA as a function of the incident power density, the modulation depth of SA is ≈20%, and the saturable intensity is ≈5.07 MW cm^−2^. It shows that the Si‐SA has a strong pulse intensity modulation ability due to the Dirac property of silicene. The laser achieves a self‐starting mode‐locking operation when the pump power reaches 145 mW. When the pump power gradually increases to 320 mW, the mode locking of the pulse reaches a stable state. The spectrum of the soliton state has a center wavelength of 1531.48 nm and a 3‐dB bandwidth of 2.84 nm (Figure 8b). According to the oscilloscope trace, there is a soliton pulse with a propagation period of 298.3 ns, a fundamental frequency rate of 3.35 MHz, and a corresponding total cavity length of 61.37 m. The pulse width is 937 fs. Therefore, the pulse's TBP is about 0.34, indicating that the light pulse is slightly chirped. The spectrum analyzer result is 43 dB. The output power is linearly related to the pump power. The maximum average output power and pulse energy are 1.078 mW and 0.322 nJ, respectively.

Haoran Mu et al.^[^ ^37^ ^]^ proposed for the first time a mode‐locked laser that uses a 10–18 L Ge nanosheet grown on top of Ge(111) and couples the SA device to the laser through a fiber‐coupled circulator. The modulation depth of Ge‐SA is ≈13.9%, the saturable intensity is ≈2.266 GW cm^−2^, and the nonsaturable loss is ≈11.90%. The linear band structure of germanene makes it a physically fast SA. It exhibits ultrafast carrier dynamics and typical pulse characteristics. A stable soliton mode‐locked state in the fiber laser cavity is observed at 1550 nm. With a pump power of 120 mW, there is a typical mode‐locked spectral feature with a center wavelength of 1569.7 nm and a 3 dB bandwidth of 3.2 nm, and the output power is ≈0.5 mW. Symmetrical Kerr sidebands indicate that the fiber laser works in a soliton mode‐locked state. The pulse sequence that is not modulated at the top indicates that the mode‐locked output quality is very high. The repetition frequency is 4.8 MHz, and the pulse duration is ≈901 fs. The TBP is ≈0.34, indicating that the chirp of the soliton pulse is small and the stability is good. The high SNR of 60 dB measured by the radio frequency (RF) spectrum further verifies the stable state of the fiber laser. In a fixed polarization state, the soliton pulse will split by increasing the pump power and behave as multiple soliton pulses.

Few layers of silicene/germanene exhibit ultrafast carrier dynamics and large optical nonlinearity. Due to the linear band structure, it is a fast SA from a physics point of view. The stable output of silicene/germanene SA mode‐locked lasers underpins the future application of Dirac materials in nonlinear optics and ultrafast photonics.

### 5.3 FB Materials‐Based SAs

Initially, FB energy band was discovered from a special type of lattice model.^[^ ^120^ ^]^ The research focuses on the superconducting physical effects of FB superlattices. In photonics, researchers reported that photonic crystals with different structures could also realize FB.^[^ 68, 121 ^]^ However, there is a lack of in‐depth research on the ultrafast dynamic process and NLO properties and applications of FB structures.

Xiaoli Sun et al.^[^ ^113^ ^]^ proposed a Yb:KYW mode‐locked laser based on 2D 3R‐NbS_2_ SA, with a pulse duration as short as 302 fs and a continuous‐wave mode‐locked (CWML) spectrum centered at 1050.6 nm with FWHM of 3.02 nm. The SNR of the base peak at 41.55 MHz reaches 53 dB, the calculated TBP is 0.36, and the pulse has a slight chirp. In a 2 h stability test, the average output power jitter was as low as 1.6%. The maximum average output power is 0.53 W.

Guanyu Liu et al. demonstrated a fiber laser spliced into a ring cavity by placing 2D Bi‐2212 SA on the cross section of the fiber pigtail.^[^ ^28^ ^]^ The laser generates noise‐like pulses with low stability. This mode‐locked state can only be maintained for about 30 min, which may be caused by instability caused by thermal effects. In the case of 2D Bi‐2212 SA with integrated SPF, in the proper polarization state, the fiber laser will start automatically when the pump power is increased to 45 mW. The output pulse period is ≈315 ns, the repetition frequency is ≈3.17 MHz, and the corresponding cavity length is ≈64.8 m. With 1559.5 nm as the center and an FWHM of 2.1 nm mode‐locked pulse spectrum, it can be observed that the output spectrum is triangular with symmetric Kelly sidebands, which indicates that it is a stable soliton state (Figure 8c). The SNR of the output pulse's RF spectrum is as high as 61.9 dB. In the intensity AC trajectory of the pulse, Sech^[^ ^112^ ^]^ fits a pulse width with an FWHM of 3.24 ps. In addition, a clear basic envelope indicates that the laser is in a noise‐like mode‐locked state. The pulse duration is 1.43 ps. Due to the slight chirp of the output pulse, the TBP is 0.37. Continuously monitoring the output pulse spectrum for more than 4 h indicates that the laser output is stable.

The band structures of 2D 3R‐NbS_2_ and 2D Bi‐2212 are both FB and have superconductivity. The experiments prove that FB materials were effective SAs in a mode‐locked fiber laser. The research on SA properties of FB materials may develop more ultrafast and NLO applications. It will arouse general interest in designing novel 2D FB materials/structures for electronic and photonic devices. It is conducive to developing novel SA devices, all‐optical switches, and other ultrafast optoelectronic devices.

One of the representative Dirac materials is graphene which has zero bandgaps with linear energy–momentum dispersion. Therefore, the electrons in graphene travel much faster than those in typical semiconductors. The optical properties of a zero‐gap system at low frequencies can be tuned or enhanced by spintronic effects. Around the K point, the energy dispersion is isotropic, and momentum k corresponds to the energy of the laser field. This leads to a node in the optical transition, arising from the fact that if the light is polarized perpendicular to the electron wave vector, no absorption is possible because of the linear energy dispersion. The spin–orbit interaction can remove this restriction because the energy is no longer strictly proportional to k. The total absorption in the important terahertz frequency range can be enhanced by up to 100%. In addition, the linear dispersion of the Dirac electrons enables broadband applications. Broadband nonlinear photon emission arises from the recombination of a distribution of nonequilibrium electrons and holes, generated by rapid scattering between photoexcited carriers after optical excitation.^[^ ^122^ ^]^ The linear dispersion of the Dirac electrons in graphene offers an ideal solution: for any excitation there is always an electron–hole pair in resonance. The ultrafast carrier dynamics,^[^ 123, 124 ^]^ combined with large absorption and Pauli blocking, make graphene an ideal ultrabroadband, fast SA. For materials with FBs at the Fermi level in its electronic structure, due to the presence of FBs, the kinetic energy is quenched and interaction‐driven quantum phases prevail. Massive carriers in FBs with hole wave functions significantly reduce the screening and enhance the exchange interaction. Compared with traditional semiconductors, FB materials show excellent ultrafast carrier dynamics.

More performance data for these three types of 2D materials‐based SAs used in ultrafast lasers are summarized in **Table** **3**. According to the above discussions and the laser characteristics listed in this table, we can conclude that fiber lasers based on these 2D materials have a high SNR and excellent stability with a slight chirp.

**Table 3:** Summary of ultrafast lasers enabled by 2D material‐based SAs

Type	Material	Gain medium	*λ* [nm]	*τ* [fs]	*f*_rep_ [MHz]	SNR [dB]	TBP	References
Semiconductor	BP	Er:fiber	1561.7	882	5.47	67	0.325	[114]
	Perovskite	Er:fiber	1555	661	13.15	61	0.41	[119]
Dirac	Silicene	Er:fiber	1531.48	937	3.35	43	0.34	[38]
	Gemanene	Er:fiber	1569.7	901	4.8	60	0.34	[37]
FB	3R‐NbS_2_	Yb:KYW	1050.6	302	41.55	53	0.37	[113]
	Bi‐2212	Er:fiber	1559.5	1.54 ps	3.17	61.9	0.41	[28]

## 6 Conclusion and Perspective

SA is a core device for realizing passively mode‐locked lasers and other extended ultrafast photonic applications. Due to the natural advantages of 2D materials, 2D materials‐based SAs have been extensively developed in the past ten years, but there are still many challenges at different aspects on the way to practical and industry‐level applications. In particular, graphene has a low modulation depth and a large unsaturation loss. BP is very unstable and will easily degrade in the atmosphere. The preparation of high‐quality and large‐area TIs and TMDs is complicated and nontrivial. These limit the use of the nonparabolic band 2D materials for wider applications in optoelectronic and photonic devices. In this regard, modifying existing SAs or exploring new SAs are the way out and long‐term goals.

As new 2D nonparabolic band materials, Dirac materials and FB materials are becoming more alluring due to their excellent optical and photoelectric properties. This review discussed the electronic band structure of semiconductor materials, Dirac materials, and FB materials and their NLO properties, such as ultrafast carrier dynamics and NLO response. Both Dirac and FBs have inherently a nontrivial band topology and may arise in certain lattices with special symmetry, such as 2D kagome and Lieb lattices.^[^ 56, 125, 126, 127 ^]^ The linear dispersive Dirac bands have massless electrons like a photon, while the nondispersive FBs host electrons with an infinite mass. The completely quenched electron kinetic energy in a FB renders naturally a strongly interactive and correlated quantum system.^[^ ^128^ ^]^

Then, we reviewed SAs based on these three types of materials and ultrafast photonics applications in mode‐locked lasers. By applying the time‐stretch dispersive Fourier transform (TS‐DFT) technique on carbon nanotubes‐based soliton laser, Liu et al. not only revealed the transition dynamics from Q switching to mode locking through four stages (initial spontaneous noise, Q switching, beating dynamics, and mode‐locking), but also real time observed that the birth dynamics of a stable SM experiences consists of five evaluation stages, i.e., the raised relaxation oscillation (RO) stage, beating dynamics stage, transient single‐pulse stage, transient bound state, and finally the stable bound state.^[^ 129, 130 ^]^ It is noteworthy that metallic carbon nanotube has two Dirac cones as that of graphene and they may share similar characteristics for ultrafast photonics.

The nonlinear optics research on Dirac materials and FB materials brings new opportunities to the 2D materials territory and provides new insights to the optical research of those unconventional materials. As interlayer coupling of the same or different 2D materials will modify the band structures, the fabrication of heterostructures with controlled twist angles may bring new family members to Dirac materials and FB materials. Though the application of Dirac materials and FB materials for ultrafast photonics is still in its infancy, we envision that those new materials will find more promising applications in the future.

## Conflict of Interest

The authors declare no conflict of interest.
